# Patient Co-Creation Initiatives in the Ambulatory Care Setting during COVID-19: A Systematic Review

**DOI:** 10.3390/medicina60010111

**Published:** 2024-01-07

**Authors:** Cristian Lieneck, Gerardo Pacheco, Mallory Cole, Liberty Hipp, Gabbie Leal, Kevin Matamoros, Brianna Rojas-Trejo, Nysa Stepp, Christian Torres

**Affiliations:** School of Health Administration, Texas State University, San Marcos, TX 78666, USA; gjp46@txstate.edu (G.P.); zkf5@txstate.edu (M.C.); xxt2@txstate.edu (L.H.); gal81@txstate.edu (G.L.); kam458@txstate.edu (K.M.); bar230@txstate.edu (B.R.-T.); nas143@txstate.edu (N.S.); exi2@txstate.edu (C.T.)

**Keywords:** co-creation, ambulatory care, outpatient, COVID-19, global pandemic

## Abstract

*Background and Objectives:* The COVID-19 pandemic has led to significant changes in ambulatory care to meet new healthcare demands. *Materials and Methods:* A review of 21 articles focusing on patient co-creation initiatives during the pandemic shows that integrating patient feedback was crucial in transforming care delivery. *Results:* Joint efforts between healthcare professionals and patients led to new patient-focused telemedicine platforms, more efficient appointment systems, and improved safety measures. These adaptations overcame care barriers and maintained continuity of care. Key themes identified include monitoring community health standards, combining technology with patient–provider communication, and enhancing patient participation in health research. *Conclusions*: These co-creation efforts not only boosted patient satisfaction and outcomes but also demonstrated the potential for long-term healthcare innovations beyond the pandemic. The review further illuminates that co-creation in healthcare, particularly in tracking community health trends, is a practical strategy that involves diverse stakeholders in shaping healthcare delivery. The widespread adoption of co-creation in outpatient care during the pandemic highlights its role in driving patient-centered behavioral changes through innovative methods like crowdsourcing and dialogue conferencing. The review also recognizes that co-creation has been instrumental in responding to demographic changes, enhancing resources, creativity, and problem-solving in municipal-volunteer collaborations. Additionally, the evolution of technology in patient–provider communication, from initial resistance in the 1990s to its current critical role, particularly during the COVID-19 pandemic, underscores its importance in enhancing healthcare service delivery and patient data communication. The review also emphasizes the need for ethically and accessibly designed technology, especially for vulnerable groups, and highlights the significance of patient involvement in healthcare research, advocating for user-centered design and shared decision-making to create truly patient-centric interventions.

## 1. Introduction

The marketing term “co-creation” refers to the collaborative effort of a company and its customers (or other stakeholders) to create valuable products, services, or experiences [1]. Patient co-creation is a pivotal aspect in enhancing the healthcare service experience, revolutionizing the traditional patient–provider dynamic [2,3,4]. Involving patients in decision-making empowers them to actively participate in their treatment plans, fostering a sense of ownership and responsibility for their health. This collaborative approach leads to increased treatment adherence and better health outcomes [2,3,4]. Secondly, co-creation ensures that healthcare services are tailored to meet individual patient needs and preferences, promoting patient-centered care and enhancing overall satisfaction [3,4].

Patient co-creation initiatives, prior to the COVID-19 pandemic, were crucial in enhancing healthcare services by actively involving patients in the design and development of medical treatments and care processes. These initiatives fostered a more patient-centered approach in healthcare, ensuring that services and treatments were more closely aligned with patients’ needs and preferences. By leveraging the insights and experiences of patients, co-creation initiatives significantly contributed to the improvement of healthcare quality and outcomes, demonstrating the value of patient input in shaping effective healthcare solutions. Further, patient engagement in the design and improvement of healthcare services allows for valuable insights and perspectives, enabling providers to identify areas for enhancement and optimize service delivery. Co-creation builds trust and strengthens the patient–provider relationship, fostering open communication and empathy [5]. Ultimately, this patient-centric approach has not only demonstrated better health outcomes/results but has also contributed to a more efficient and sustainable healthcare system by reducing the likelihood of unnecessary procedures or treatments [2,3,4].

During the COVID-19 pandemic, the importance of patient co-creation of their healthcare experience has been magnified, becoming a critical factor in navigating the challenges brought on by the global health crisis [4]. Involving patients in decision-making during this unprecedented time empowers them to actively participate in their care, considering their unique circumstances and concerns. With COVID-19 affecting individuals differently based on various factors like age, pre-existing conditions, and socio-economic status, co-creation ensures that healthcare services are personalized and responsive to each patient’s specific needs [4,5].

Patient co-creation also plays a significant role in promoting adherence to public health guidelines and safety protocols. By engaging patients in the decision-making process, healthcare providers can better explain the importance of preventive measures, such as wearing masks, practicing social distancing, and getting vaccinated [3,5]. Patients who are involved in these decisions are more likely to understand the rationale behind these measures, which can lead to higher compliance rates and a collective effort to curb the spread of the virus.

The pandemic has also highlighted the need for flexibility and agility in healthcare delivery [5]. By actively involving patients in the design and improvement of healthcare services, providers can better adapt to the rapidly changing circumstances caused by COVID-19. This approach allows healthcare systems to remain responsive to patient feedback and rapidly implement necessary changes to enhance patient experiences and overall outcomes. Additionally, the emotional toll of the pandemic has been immense, with many patients facing isolation, anxiety, and fear. Patient co-creation fosters a more empathetic and understanding healthcare environment. By involving patients in decision-making, healthcare providers can address not only the physical but also the emotional and mental health needs of individuals during these challenging times [1,2,5]. This patient-centric approach not only improves the quality of care but also strengthens the patient–provider relationship and builds trust. Additionally, the pandemic has driven a surge in telemedicine and remote healthcare services [5]. Patient co-creation becomes vital in this context as it ensures that these virtual interactions are tailored to suit individual patient preferences and accessibility requirements. By actively seeking patient feedback on telemedicine experiences, healthcare providers can identify technological barriers, improve user interfaces, and enhance overall telehealth services [5].

Patient co-creation of their healthcare experience during the COVID-19 pandemic is crucial for delivering patient-centered care, promoting adherence to safety measures, fostering flexibility in healthcare delivery, addressing emotional needs, and optimizing telemedicine services. It not only empowers patients to actively participate in their care but also strengthens the healthcare system’s response to the ongoing challenges posed by the pandemic. By placing patients at the center of decision-making, healthcare providers can forge a stronger alliance to combat COVID-19 and cultivate a more resilient and patient-focused healthcare system for the future.

This rapid review focuses on the identification of patient co-creation initiatives identified in the published literature during the COVID-19 pandemic. The research team’s primary initiative was to potentially identify any similarities of co-creation initiatives prior to COVID-19, while also identifying any potential new co-creation efforts and their effect(s) on stakeholder cost, quality, and access to care. This review is unique in this regard, as no prior rapid reviews (or full systematic literature reviews) have been conducted to date utilizing these aggressive search parameters specific to the smaller-scale marketing initiative of co-creation for the healthcare industry.

## 2. Methods

This systematic review adhered to the PRISMA (Preferred Reporting Items for Systematic Reviews and Meta-Analysis) guidelines, and the review was registered on PROSPERO (ID # 471424). The research team sourced literature concerning (ambulatory care outpatient) provider organizations from four databases: CINAHL Complete, Academic Search Ultimate, Business Source Ultimate (via EBSCO host), and PubMed (which accesses MEDLINE). The decision to utilize four databases was motivated by a preliminary observation of scarce publications fitting our search parameters. Notably, peer-reviewed articles focusing on the aspects of healthcare service marketing, specifically co-creation initiatives in the ambulatory care segment of the industry, are sparse, emphasizing their importance as the ongoing transition to postpandemic routine operations endures. The study concentrated on outpatient organizations and the co-creation concepts, terms, and actions performed and/or described by the research articles within the study’s associated outpatient care organizations. The National Library of Medicine’s Medical Subject Headings (MeSH) thesaurus, used to index articles for PubMed (MEDLINE), helped pinpoint essential search terms. We employed Boolean operators to guarantee accurate word/phrase inclusion based on MeSH terms and further extended search terms specific to ambulatory care organizations. The following search string was used in the search with one term truncated (*) to also include plural versions of the search term:

[(“ambulatory care” OR “outpatient care” OR “outpatient services” OR “urgent care” OR “clinic visit*”) AND (“cocreation” OR co-creation” OR “collaborative” OR “collaboration” OR “shared creation” OR “participation” OR “participative”)]

### 2.1. Inclusion Process

The review articles were included in the study if they were published between 1 January 2015 and 1 June 2022. This date range was decided upon by the research team to ensure that a sufficient number of articles were identified by the search string supporting the research topic, while also obtaining recent healthcare marketing initiatives related to co-creation in the outpatient setting. Only peer-reviewed publications were utilized in the review, as well as a full-text limitation so that follow-on full manuscript screenings and analyses could occur.

The information for this study was derived from secondary data sources, specifically a library research database. The literature incorporated into this research was accessible to the public, and individual research subjects, if any, remained anonymous. Consequently, this systematic review meets the criteria for an “exempt” status as per 45 Code of Federal Regulations (CFR) 46. There was no need for an institutional review board assessment, and obtaining consent was not applicable.

### 2.2. Exclusion Process

**Figure 1:**
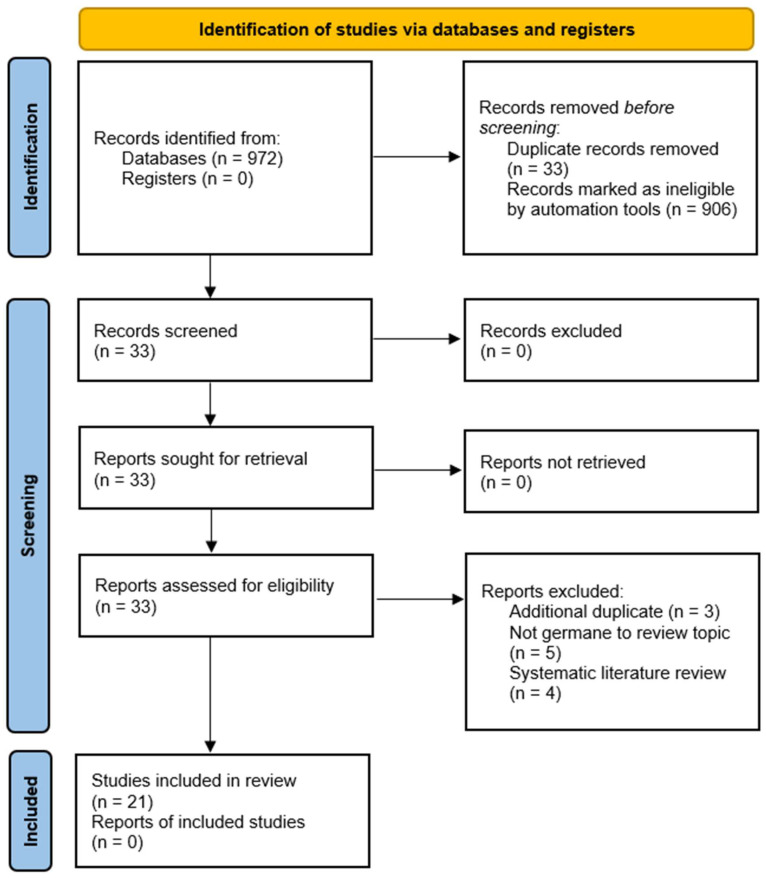
Preferred Reporting Items for Systematic Reviews and Meta-analysis (PRISMA) figure that demonstrates the study selection process.

The article exclusion process (Figure 1) demonstrates the identification, screening, and exclusion of articles from the beginning to the end of the search process. In the end, 21 articles were identified in the review as meeting the search criteria. Beginning with 972 articles initially identified in the research databases, 33 articles were immediately (and automatically) removed from the search as duplicated by the EBSCO host engine. Further, 906 articles were then removed using the EBSCO host site’s filtering options set to full-text only, English only, and peer-reviewed only.

Because only 33 articles remained in the review process, the research team did not conduct abstract screening of the manuscripts and instead went directly to the full article reading/analysis. Table 1 demonstrates the division of reading and analysis conducted by the research team, with each article being reviewed by at least two members of the research team, per PRISMA guidelines.

Upon completion of the full manuscript review by the research team members, three articles were removed as additional duplicates, five articles were identified as not germane to the research topic (erroneously identified by the database search engines), and four manuscripts were removed for being classified as literature and/or systematic reviews focused on other areas of ambulatory care management and associated concepts outside the scope of this review.

## 3. Results

Table 2 provides a summary of findings by the research team for each article included in the review (n = 21). Basic article information is provided, as well as the patient and/or population segment investigated in the study, and summary comments of the study’s overall purpose and co-creation initiatives and outcomes identified by the research team in their article analysis.

The study designs employed in the reviewed column of Table 2 were diverse and tailored to the specific aims of each healthcare intervention, with a strong emphasis on co-creation methodologies. These ranged from single-arm studies and randomized controlled trials to participatory design, experience-based codesign, community-based participatory research, and qualitative program evaluations, all converging on the development, assessment, and enhancement of healthcare applications and interventions.

**Figure 2:**
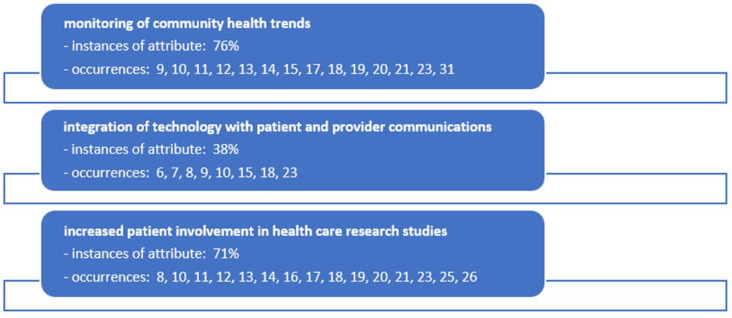
Occurrences of underlying themes (constructs) identified in the literature for patient co-creation in the ambulatory care setting during the COVID-19 pandemic.

The research team conducted a thorough analysis through a series of webinar and in-person meetings to distill key themes from the literature on patient co-creation in healthcare. The first theme identified was the “monitoring of community health trends”, which emerged as a predominant topic in 76% of the selected literature, highlighting its importance in understanding and responding to public health needs. The second theme focused on the “integration of technology with patient and provider communications”, which was featured in 38% of the literature, reflecting a growing emphasis on leveraging digital tools to enhance healthcare delivery and patient engagement. Lastly, “increased patient involvement in healthcare research studies” was a significant theme, with a 71% occurrence, underscoring a shift towards more inclusive research practices that value patient experiences and insights. These themes collectively represent a multifaceted approach to patient co-creation, illustrating the dynamic interplay between community health monitoring, technological advancements, and participatory research in crafting a patient-centered healthcare landscape. The research team’s analysis of the articles identified in the review are identified in Figure 2.

## 4. Discussion

The review team’s analysis of findings from the literature on patient co-creation initiatives, particularly within the scope of monitoring community health trends, integrating technology in patient–provider communications, and enhancing patient involvement in healthcare research, demonstrate a unique application of patient co-creation initiatives identified during the COVID pandemic. Co-creation has continued to emerge as a valuable construct in public and community health, as evidenced by its prevalence in the literature, with a notable focus on the pandemic’s outpatient care. Crowdsourcing and dialogue conferencing are highlighted as key strategies for gathering diverse stakeholder input, while the Community Health System Innovation model (COHESION) model is presented as a paradigm for embedding patient engagement deeply into the healthcare process. The section also touches on the potential of interdisciplinary approaches to co-creation, like healthcare ecosystem mapping, to illuminate systemwide issues and facilitate collaborative problem-solving. Furthermore, it underscores the necessity of ethical considerations and technology’s role in co-creation, particularly in facilitating communication and data capture during the pandemic. The review also identified the transformative power of co-creation in healthcare research, which can lead to more patient-centered care and improved health outcomes.

### 4.1. Monitoring of Community Health Trends

The use of co-creation in public and community health was identified by the research as a construct within the identified literature with the highest percentage instance of attributes in the review (76%). The research team identified several subconcepts demonstrating the use of co-creation in outpatient care during the pandemic, with several elements identified and discussed below.

Crowdsourcing serves as a mechanism to co-create improvements in healthcare by gathering ideas and information from various stakeholders such as patients and healthcare providers [12]. Crowdsourcing, when combined with mobile health interventions, has the potential to catalyze patient-centered behavioral changes. Despite the similarities, dialogue conferencing is distinct from crowdsourcing. It involves convening diverse stakeholder groups to collaborate and exchange knowledge, with a focus on devising potential future solutions [12].

In Norway, the challenge of an ageing population and declining birth rates have led to a shift in government policies. These policies now encourage collaboration between municipalities and volunteers, emphasizing coproduction as a method to harness resources effectively [8]. This collaboration can amplify resources and stimulate creativity through diversified dialogues and problem-solving, provided the assumptions, attitudes, and beliefs of each partnering entity are considered [13].

When considering the integration of co-creation in healthcare, the COHESION model stands out [20]. This co-creation method underscores the pivotal role of patient engagement throughout the healthcare delivery process, from design and implementation to evaluation. Techniques like focus groups and interviews act as platforms for discussions, idea exchanges, and capturing insights about pertinent health issues [20]. When stakeholders actively participate in the healthcare system, they often feel a greater sense of accountability, propelling them towards actions that improve community health [20].

The interdisciplinary landscape has recognized the significance of co-creation. An example of this is an interdisciplinary team that employed a multimethod co-creation approach to devise a healthcare ecosystem map. This tool was instrumental in presenting a comprehensive view of the healthcare system, pinpointing areas of concern, and outlining requisite care components [16]. In the realm of healthcare, this type of design thinking has emerged as an influential problem-solving tool. Once an issue is identified, this method facilitates the brainstorming of potential solutions. When applied to healthcare trends, design thinking, buoyed by principles of co-creation and coproduction, can spotlight inefficiencies and reveal opportunities for transformative changes through stakeholder engagement.

The literature increasingly indicates that co-creation can elevate efficiency, patient satisfaction, trust, and research potential [9,12,16,17,20]. For instance, primary care interventions hold the promise of reducing involuntary psychiatric admissions, but there is a void in such interventions. To bolster quality and innovation, it is essential to incorporate insights from external stakeholders and individuals with firsthand experience [9,10,21,26]. One of the pressing issues in medical research is ensuring ethical governance over data sharing, which necessitates leveraging social licenses. Chronic obstructive pulmonary disease (COPD), for instance, can benefit from a culturally sensitive, co-created healthcare map, offering a more holistic understanding of the disease.

Keeping pace with technological advancements, the integration of electronic nicotine delivery systems (ENDS) is gaining traction in family medicine practices [14]. Initiatives like citizen-science projects, emphasizing the monitoring of environmental factors like air and noise pollution, underscore the importance of sustainable research endeavors. Such monitoring techniques are also instrumental in providing clarity on challenges and opportunities for researchers aspiring to co-create interventions in low-resource settings [14,18]. Finally, the rising trend of living labs offers valuable insights, particularly for healthcare providers catering to dementia patients, helping determine the most suitable living arrangements for them, be it community living or long-term facilities [14,18].

### 4.2. Integrating Technology in Patient/Provider Communications

In the 1990s, co-creation initiatives in home health organizations faced challenges, primarily due to poor implementation and resistance to digital health technology. However, technology holds great promise in healthcare, notably in minimizing preprocedural stress and anxiety. This is particularly relevant in moments leading up to and during outpatient visits [10]. Distance monitoring has emerged as a pivotal component of co-creation, with physicians and nurses spearheading the identification of suitable patients and offering technical support [10]. Especially during the COVID-19 pandemic, technology played a vital role, as seen with applications designed to capture patients’ health data and communicate them to healthcare providers [10].

It is essential that technological integrations consider accessibility and ease of use, especially keeping disability services in mind to ensure applications are legible and functional. Ethical concerns arise when dealing with patients, such as those with dementia, who may not fully grasp the technology’s implications [6,10,14,20]. To effectively merge technology with patient care, continuous, innovative research, characterized by validity and a robust methodology, is crucial.

### 4.3. Patient Involvement in Healthcare Research Studies

Co-creation in healthcare research augments the quality-of-care delivery and can have a positive influence on patient health outcomes [6,8,15,18]. By fostering collective collaboration among stakeholders, particularly patients, the healthcare system can gather crucial data, formulate new strategies, and shape public health discussions [21,23,26]. The primary objective of co-creation is to render healthcare services more patient-centric [8,9]. This involves researching issues, devising and enacting interventions, and defining outcomes in collaboration with patients, family carers, professionals, researchers, and other key stakeholders.

One approach integral to co-creation’s success is user-centered design, which emphasizes involving end-users in system design and decision-making processes. For instance, a research study highlighted how electronic health records (EHRs) were underutilized. Through co-creation, the study revealed the potential of EHRs to efficiently document patients’ interactions with electronic nicotine delivery systems (ENDs). In another study focused on chronic diseases like COPD and heart failure (HF), co-creation was employed to create an ecosystem map detailing patient care needs and existing healthcare services [16,25].

Furthermore, shared decision-making exemplifies co-creation in practice, allowing patients to have a more active role in their care, resulting in improved understanding and reduced decisional conflicts [24]. The overarching principle behind co-creation is fostering a collaborative mindset where stakeholders or end-users jointly produce outcomes beneficial to all. Citizen science, another facet of co-creation, empowers the general public to play a role in scientific endeavors, bridging the gap between science and society. This collaborative approach has been shown to improve patient comprehension of medications and boost their confidence, knowledge, and skills in healthcare contexts.

## 5. Conclusions

In light of the challenges posed by the COVID-19 pandemic, co-creative patient endeavors in ambulatory care have illustrated significant progress in several areas, as highlighted in this swift literature analysis. One key observation is the enhanced capability to uphold and even improve community health standards, a testament to the adaptability and resilience provided by co-creation during widespread health emergencies.

The comprehensive review findings elucidate that co-creation in healthcare, particularly in the monitoring of community health trends, is not merely a theoretical construct but a pragmatic approach that actively engages various stakeholders in shaping healthcare delivery. The high prevalence of co-creation in outpatient care during the pandemic underscores its potential for fostering patient-centered behavioral changes through innovative strategies like crowdsourcing and dialogue conferencing. In Norway’s response to demographic shifts, co-creation has facilitated resource amplification, creativity, and effective problem-solving in municipal-volunteer collaborations. The COHESION model and interdisciplinary healthcare ecosystem mapping are highlighted as exemplary methods that engage patients across the healthcare delivery spectrum, from design to evaluation, encouraging accountability and participation in community health. Additionally, technology’s integration into patient–provider communications has evolved from resistance in the 1990s to becoming a cornerstone of contemporary healthcare, particularly during the COVID-19 pandemic, enhancing the delivery of healthcare services and patient data communication. The review also emphasizes the importance of ethical and accessible technology design, especially for vulnerable populations. Lastly, patient involvement in healthcare research, through user-centered design and shared decision-making, has been pivotal in creating interventions that are truly patient-centric, improving care quality, and empowering patients in healthcare decision-making processes.

Like any systematic literature review, this research does present with limitations. The aggressive search parameters (specifically, the publication date range) to control for publications occurring during the global pandemic yielded a low number of articles for review. As a primary search requirement, co-creation initiatives related to the COVID-19 global pandemic were identified using the publication date range of the articles, as identified by the EBSCOhost platform. Therefore, some articles identified did not specifically reference COVID directly, yet still met the review’s search criteria. It was found that some articles identified were located in journals not in Scopus or Scimago, potentially questioning their scientific validity. Additionally, the use of the initiative “co-creation” was broadened using synonyms as suggested by the EBSCO host library website. Further examples of co-creation may be present in additional healthcare marketing articles without being captured by this review’s chosen search string. The research team also identified two important areas for future research coming from this review effort: discussion and limitations on biases (for example, the user-centered approach and the generalizability to other populations), and how different user groups adapted to the technology (and potential health literacy challenges) during the pandemic.

The reinforced synergy between technology and patient–healthcare provider interactions, championed by co-creative approaches, has undeniably transformed healthcare experiences, emphasizing promptness, efficiency, and broader accessibility. In a period marked by the ubiquity of digital communication, such progress underscores the importance of tech-integrated solutions. Additionally, the notable uptick in active patient participation in healthcare research underscores a shift towards a more holistic research approach that centralizes patient voices, experiences, and feedback. Together, these findings highlight the potential of co-creation to redefine ambulatory care, particularly during challenging times.

## Figures and Tables

**Table 1:** Reviewer assignment of initial database search findings (full article review).

Article Assignment	Reviewer 1	Reviewer 2	Reviewer 3	Reviewer 4	Reviewer 5	Reviewer 6	Reviewer 7	Reviewer 8	Reviewer 9
**Articles 1–10**	X	X	X				X	X	X
**Articles 11–21**	X	X	X	X	X	X		X	X

**Table 2:** Summary of findings (n = 21).

Reference Number and Authors(s)/Year	Article Title	Journal/Publication	Participant/Population and Location	Purpose/Method	Co-Creation Outcome/Observation
[6] Poot et al., 2023	How to use participatory design to develop an eHealth intervention to reduce preprocedural stress and anxiety among children visiting the hospital: The Hospital Hero app multi-study and pilot report	*Frontiers in Pediatrics*	Children undergoing medical procedures and their caregivers. The Netherlands.	To assess the development and use of the “Hospital Hero” eHealth app. Single-arm study; multistudy and pilot report. Participatory design to develop an eHealth intervention—The Hospital Hero app.	Decrease in preprocedural stress and anxiety, app usability, user-experience, feasibility, qualitative insights into user opinions and experiences. Reduction of preprocedural stress and anxiety.
[7] Moser and Korstjens, 2022	Series: Practical guidance to qualitative research. Part 5: Co-creative qualitative approaches for emerging themes in primary care research: Experience-based co-design, user-centered design and community-based participatory research	*The European Journal of General Practice*	Primary care patients, family carers, researchers, care professionals, and other relevant stakeholders. In particular, hard-to-reach and vulnerable individuals for community-based participatory research. The Netherlands.	Three co-creative approaches: (1) Experience-based codesign, (2) User-centered design, (3) Community-based participatory research. Standard primary care research methods without co-creative characteristics served as the control group.	Practical guidance. Definition of (research) problems, development and implementation of interventions, evaluation and defining (research and practice) outcomes. Understanding of healthcare processes, design of technological and organizational systems, and addressing locally relevant health issues.
[8] Martens et al., 2022	Practical guidance to qualitative research. Part 5: Co-creative qualitative approaches for emerging themes in primary care research: Experience-based co-design, user-centered design and community-based participatory research	*BMJ Open*	n/a.	Process evaluation of the intervention scale-up.	Study protocol for integrated diabetes and hypertension care.
[9] Samper-Pardo et al., 2023	Development and Validation of a Mobile Application as an Adjuvant Treatment for People Diagnosed with Long COVID-19: Protocol for a Co-Creation Study of a Health Asset and an Analysis of Its Effectiveness and Cost-Effectiveness	*International Journal of Environmental Mental Research and Public Health*	People diagnosed with long COVID-19. Spain.	Randomized clinical trial with two groups: (1) control group receiving TAU, and (2) interventional group receiving TAU + APP + MI. Evaluations at baseline, three, and six months postintervention. Mobile application (APP) as a community health asset (HA) with recommendations and recovery exercises, along with three motivational interviews (MI). Effectiveness and cost-effectiveness of the application.	Mobile application as an adjuvant treatment. Overall effectiveness and cost-efficiency of the APP. Improvement in quality of life, number and severity of ongoing symptoms, physical and cognitive functions, affective state, and sleep quality.
[10] Enam et al., 2022	Impact of distance monitoring service in managing healthcare demand: a case study through the lens of cocreation	*BMC Health Services Research*	Impact of distance monitoring service. Patients with chronic conditions in a primary healthcare setting in Norway.	Case study. Implementation of distance monitoring services in the healthcare delivery process.	Distance monitoring service in managing healthcare demand. Changes in care delivery processes, activities, channels of interaction, roles of healthcare providers, and potential impacts on healthcare demand. Potential for better demand management and complexities introduced. Mechanisms for reducing these complexities.
[11] Marshall-McKenna et al., 2023	A multinational investigation of healthcare needs, preferences, and expectations in supportive cancer care: co-creating the LifeChamps digital platform	*Journal of Cancer Survivorship: Research and Practice*	Individuals in need of supportive cancer care. Cancer survivors diagnosed with breast cancer, prostate cancer, or melanoma; adult family caregivers; and healthcare professionals involved in oncology in Greece, Spain, Sweden, and the UK.	Multinational investigation that focused on understanding the needs, preferences, and expectations in supportive cancer care and the introduction of the LifeChamps digital platform for supportive care.	The LifeChamps digital platform assessed healthcare needs, preferences, and expectations. Perceived healthcare needs, preferences, expectations, and reactions towards the proposed LifeChamps digital platform. Identified gaps in supportive care, anticipated challenges, and potential benefits of the platform.
[12] Sha et al., 2022	Co-creation using crowdsourcing to promote PrEP adherence in China: study protocol for a stepped-wedge randomized controlled trial	*BMC Public Health*	PrEP users in China; Chinese key populations at high risk for HIV.	Stepped-wedge randomized controlled trial. Co-created intervention packages developed to facilitate PrEP adherence, including social-media-based intervention materials, in addition to the standard of care. Also, use of a WeChat miniapp for sexual and mental health education.	Co-creation using crowdsourcing to promote PrEP adherence. Primary outcomes are PrEP adherence and retention in PrEP care throughout the study period. The hypothesis being tested is that a co-created intervention can enhance PrEP adherence.
[13] Wormdahl et al., 2022	The ReCoN intervention: a co-created comprehensive intervention for primary mental health care aiming to prevent in-voluntary admissions	*BMC Health Services Research*	Individuals in primary mental healthcare. Adults at risk of involuntary psychiatric admissions in Norway, with a focus on those living in the community and engaging with primary healthcare professionals.	ReCoN (Reducing Coercion in Norway) intervention developed for primary mental healthcare. This comprehensive intervention encompasses six strategy areas with multiple action areas and specified measures.	Prevention of involuntary admissions. Co-created comprehensive intervention. Prevention of involuntary psychiatric admissions in adults, and enhancement of the quality and development of primary mental healthcare services to meet stakeholders’ needs.
[14] Muller et al., 2021	The social license for data-intensive health research: towards co-creation, public value and trust	*BMC Medical Ethics*	Medical research projects that rely upon the reuse and/or linkage of health data, particularly in the context of big-data-driven health research. Incorporation of cessation in family medicine.	Qualitative program evaluation. Implementation of a social license as a guideline for ethical governance, focusing on trustworthiness and co-creation, while engaging patients and the public from the start.	Co-creating opportunities for cessation for electronic nicotine delivery systems. Preservation of public trust, recognition of a broad range of stakeholder interests, especially those of patients contributing data, and establishment of a more ethical practice of governance.
[15] van Rooijen et al., 2021	How to foster successful implementation of a patient reported experience measurement in the disability sector: an example of developing strategies in co-creation	*Research Involvement and Engagement*	Patients in the disability sector. Care-users who are communication-vulnerable, professionals, management, and researchers involved in the implementation process of patient-reported experience measures.	Participatory action research where implementation strategies were co-created by all stakeholders over 9 months, aiming for a successful integration of patient-reported experience measures. Patient-reported experience measurement.	Successful implementation strategies. Example of developing strategies in co-creation. Impact of each stakeholder group on the development of tailored implementation strategies, understandability, relevance, and the look and feel of strategies, and insights into supportive conditions for engaging communication-vulnerable care-users.
[16] Hussey et al., 2021	Confronting complexity and supporting transformation through health systems mapping: a case study	*BMC Health Services Research*	Health system stakeholders and innovators in Ontario, Canada, focusing on patients with chronic obstructive pulmonary disease (COPD) and heart failure (HF).	Health systems’ mapping case study. Co-creation of a healthcare ecosystem map using a collaborative approach that involved focus groups, open-ended questionnaires, and document review to provide a visual representation of the current health system state concerning COPD and HF.	Confronting complexity and supporting transformation. Identification of key sectoral components, intercomponent interactions, and care requirements for COPD and HF patients. Recognition of care gaps, accessibility issues, and resource distribution in the current system. Improved collective understanding of the system.
[17] van Weel Baumgarten et al., 2021	Co-creation and collaboration: a promising approach towards successful implementation. Experience from an integrated communication and mental health skills training programme for Japanese General Practice	*Patient Education and Counseling*	Japanese general practice patients. Japanese GPs participating in the integrated training program.	Integrated communication and mental health skills training program. Co-created integrated training program in communication and depression assessment and management. Includes didactic and experiential training methods, practicing and feedback, and a “train-the-trainer” component.	Co-creation and collaboration approach. Experience from an integrated program. Adoption and continuation of the training elements in daily practice, teaching communication and depression management skills, and positive evaluation of training content, methods, and the participatory approach.
[18] Kovach et al., 2021	Co-creating opportunities to incorporate cessation for electronic nicotine delivery systems in family medicine—a qualitative program evaluation	*BMC Family Practice*	Family medicine practices in the U.S. dealing with electronic nicotine delivery systems (ENDS) cessation.	Incorporation of cessation in family medicine. Qualitative program evaluation. Incorporating ENDS cessation into the practice using a co-created approach informed by the collective knowledge of participants, expert consultants, and the research team.	Co-creating opportunities for cessation for electronic nicotine delivery systems. Identification of opportunities to improve ENDS cessation in three categories: leading change, creating processes, and assisting patients who use ENDS. Positive changes in practices’ ability to handle ENDS cessation based on the outlined opportunities.
[19] Froeling et al., 2021	Narrative review of citizen science in environmental epidemiology: Setting the stage for co-created research projects in environmental epidemiology	*Environment International*	Environmental epidemiologists; citizens involved in co-created projects	The implementation of citizen-science (CS) projects in environmental epidemiology, particularly co-created projects Narrative review of citizen science in environmental epidemiology.	Co-created research projects in environmental epidemiology. Enhanced understanding and application of CS in environmental epidemiology. Democratization of scientific governance. Sustainability of research projects. Development of locally relevant research designs. Effective use of local knowledge. Obtaining medical ethical clearance. Co-analysis of exposure and health associations. Increased outreach activities.
[20] Lazo-Porras et al., 2020	Lessons learned about co-creation: developing a complex intervention in rural Peru	*Global Health Action*	Primary healthcare settings in a low-resource setting in Peru.	Complex intervention in rural Peru. Use of the COHESION manual for co-creation to develop interventions aimed at improving diagnosis and/or management of chronic diseases.	Lessons learned about co-creation surrounding the development of a complex intervention. Successful adaptation and customization of the COHESION manual to the local context of rural communities in northern Peru. The design of a theory of change for activities included in the complex intervention. Development of specific interventions such as training for health workers, radio programs, and small grants for PHC infrastructure improvement. Insights into the co-creation process and lessons learned.
[21] van der Boog et al., 2020	A Self-management Approach for Dietary Sodium Restriction in Patients With CKD: A Randomized Controlled Trial	*American Journal of Kidney Diseases*	Patients with chronic kidney disease. Adults with CKD stages 1 to 4 or a functioning kidney transplant, hypertension, and sodium intake >130 mmol/d attending nephrology outpatient clinics in 4 Dutch hospitals.	Standard dietary advice. Routine care plus a web-based self-management intervention including individual e-coaching and group meetings implemented over a 3-month intervention period, followed by e-coaching over a 6-month maintenance period.	Self-management approach for dietary sodium restriction involving dietary sodium restriction. Sodium excretion after the 3-month intervention and after the 6-month maintenance period. Blood pressure changes. Proteinuria levels. Costs associated with the interventions. Quality-of-life measurements. Self-management skills assessment. Barriers and facilitators for implementation.
[22] Nilsen et al., 2020	Implementation of eHealth Technology in Community Health Care: the complexity of stakeholder involvement	*BMC Health Services Research*	Eight municipalities in Norway.	Qualitative research. Implementation of eHealth technology in community healthcare. Implementation of eHealth technology initiatives in community healthcare.	Stakeholder involvement complexity. Identification of stakeholders involved in the implementation of eHealth. Understanding of relationships and dependencies among stakeholders. Categorization of stakeholders based on their dimensions and degree of integration (external–internal; core, support, and peripheral stakeholders). Insights into the complexity of stakeholder integration in public community healthcare.
[23] Verloo et al., 2020	A Comprehensive Scoping Review Protocol of Using Living Labs to Explore Needs and Solutions for Older Adults with Dementia. Smart Homecare	*Technology and TeleHealth*	Older adults with dementia. Older adults with dementia, either living in the community or in long-term healthcare facilities (LTHFs).	Co-creation for older adults with dementia. Using living labs to explore needs and solutions. Implementation and utilization of living labs to study and support the health, independent living, and well-being of older adults with dementia.	Using living labs to explore needs and solutions. Identification of living-lab activities and types of co-creations. Exploration of the needs and expectations of older adults with dementia. Identification of innovative products or service solutions developed in living labs to assist this population. Evaluation of the methodological quality of the studies and effectiveness of the technological products or service solutions.
[24] Pel-Littel et al., 2020	The development of the evidence based SDMMCC intervention to improve shared decision making in geriatric outpatients: the DICO study	*BMC Medical Informatics and Decision Making*	Geriatric outpatients. Older adults with multiple chronic conditions (MCC) and their informal caregivers.	SDMMCC intervention for shared decision-making. Training for geriatricians on skills to involve older adults with MCC and their caregivers in SDM, following the six-step “Dynamic model for SDM with frail older patients”. Preparatory tool for older patients and their informal caregivers to participate in SDM.	Improved shared decision-making in geriatric outpatients. Geriatricians’ skills and confidence in implementing SDM with older adults with MCC. Older patients’ and caregivers’ engagement, understanding, and satisfaction with the decision-making process. Effectiveness of the preparatory tool in facilitating SDM.
[25] Watson et al., 2020	Care home nursing: Co-creating curricular content with student nurses	*Nurse Education Today*	Student nurses from years one to four undertaking an honors bachelor’s degree in nursing.	Co-creation of curricular content on care home nursing.	Co-creation of curricular content. Attitudes of student nurses towards care home nursing. Shift in perceptions of care home nursing as a career choice. Suggestions from students for developing better curricular content and learning opportunities related to care home nursing.
[26] Andfossen, 2020	Co-production between long-term care units and voluntary organizations in Norwegian municipalities: A theoretical discussion and empirical analysis	*Primary Health Care Research and Development*	Long-term care units and voluntary organizations in Norwegian municipalities.	Theoretical discussion and empirical analysis involving coordination and collaboration in volunteer activities within care services.	Coproduction between long-term care units and voluntary organizations. Extent of collaboration between LTC units and voluntary organizations in coordinating voluntary activities. Understanding of whether they share tasks, divide tasks, or both. Efficiency and optimization of resource usage in the care sector through collaboration.

## Data Availability

Data are contained within the article.
